# Hospital and Institutional News

**Published:** 1913-06-14

**Authors:** 


### HOSPITAL AND INSTITUTIONAL NEWS.

### THE PROGRAMME OF THE OXFORD CONFERENCE.

We have pleasure in publishing the full programme of the fourth annual Conference of the British Hospitals Association, to be held at Oxford from June 26 to 28. Thanks to the kindness of the Vice-Chancellor of the University, the meetings will be held in the Examination Schools in the High Street. Sir William Osler, Regius Professor of Medicine, will act as President, and his Presidential Address will open the proceedings on Thursday morning, June 26, at 10 A.M. The morning session will be devoted to considering the effect of the Insurance Act on hospitals, with a view to its amendment. On Thursday afternoon at 2.30 Sir Thomas Oliver will deliver an address on "Tuberculosis and the General Hospitals," and a discussion will follow. At 5 P.M. a visit to the Radcliffe Infirmary will be made, and tea provided. On Friday, June 27, at 10 A.M., Mr. Keith Young, F.R.I.B.A., will read a paper on "The Upkeep of Hospitals." A discussion and questions will follow. A business meeting will then be held to elect the Council and honorary officers for the ensuing year and to receive suggestions as to the time and place of the next annual Conference. The afternoons of Friday and Saturday will be given up to visits to the Colleges and places of interest in the neighbourhood.

### THE INCOME OF THE KING'S FUND.

A most regrettable feature in the sixteenth annual report of King Edward's Hospital Fund, which has recently been issued, is the record of a serious diminution for the year under review of the total receipts, which amounted to only £179,378, as compared with £235,620 received in 1911. This is the smallest income recorded since 1906. The diminution is shown in three important items: in annual subscriptions and donations there was a fall of £8,221, though it should be remembered that the comparative figures for 1911, after excluding Sir Ernest Cassel's gift of £31,500, were inflated by an anonymous donation of £10,000; the receipts from legacies show a far more serious decrease of £37,139. In view of this decline of legacies to a point far below the average, the maintenance of a distribution of £157,500 was only made possible by drawing the sum of £18,758 from reserves resulting from the receipt in recent years of exceptionally large legacies. A very gratifying feature is the contribution of £18,000 by the League of Mercy, actually an increase of £1,000 over the sum contributed in 1911. The total sum distributed, £157,500, was the same as in 1911, apart from the supplementary distribution of £31,500 made possible in that year by Sir Ernest Cassel's gift. Of this sum £151,000 was allocated to ninety-nine London hospitals and £6,500 to forty-one convalescent homes and nine consumptive sanatoria taking London patients. The rapid growth of the Fund during the comparatively short period of its existence is indicated by its enormous accumulated funds, the amount retained on capital at the close of 1912 being £1,084,393, and on the reserve fund £746,059, after allowing for the transfer to the distribution account at the close of 1912.

### ST. GEORGE'S HOSPITAL AND THE KING'S FUND.

After we go to press a special court of the governors of St. George's Hospital is to be held—*i.e.* on the 12th inst., at 3 P.M., to consider the question of confirming the resolution of the house committee of April 30, which was referred to the decision of the next court: "That the hospital revert to the former method of keeping accounts and provide the difference between £1,000 and the sum now expended on the Bacteriological and Pathological Departments from the Discretionary Fund, in accordance with the suggestion of the King's Fund." The Superintendent, Dr. Friend, whose appointment to Christ's Hospital we note elsewhere, notifies further that Lord Moulton of Bank and Sir George Hayter Chubb have, at the request of the President of the hospital, Princess Christian, consented to form an independent committee to go into the details of the whole question at issue between St. George's Hospital and the King's Fund. We understand that at this court an opportunity will be given to learn at first hand what the policy of St. George's Hospital in this matter is.

### CAMBERWELL GUARDIANS AND CORPORAL PUNISHMENT.

The recent decision of the Camberwell Board of Guardians in approving of the corporal punishment of unruly boys in the convalescent wards of the Camberwell Infirmary has been followed by a protest meeting. Mr. W. J. Hawke moved a resolution protesting against the above decision, and calling for a Local Government Board inquiry. If, he said, patients were well enough to be whipped they were well enough to be discharged. The Countess de Lormet, the only member of the Guardians who was present, said that nothing of the sort was allowed in a hospital, and that the Guardians had tried to ignore and belittle the matter in every way. Another speaker declared that this was a matter for the public of Camberwell, and not wholly for the Guardians to determine. It is useless to try and laugh this protest meeting out of court. In our issue of May 10 we gave excellent reasons why the Medical Superintendent, Dr. Keats, was seen to be ill-advised in resorting to corporal punishment, and why the Guardians were ill-advised in approving it. Corporal punishment is upheld as a short cut to discipline; but the truth is that in matters of this kind there are no short cuts. Wherever corporal punishment is practised or sanctioned its liability to grave abuse provides an infinity of occasions for the bringing of charges and the invoking of scandals. We are afraid that the Camberwell Guardians have not heard the last of their regrettable decision, and will never hear the last of it until they have found out by experience the heavy price that they are paying for condoning this practice.

### A QUESTION OF SANATORIUM CONTROL.

The board of Winsley Sanatorium have been recently discussing whether the medical officer should be the superintendent or not. The medical men associated with Winsley are naturally anxious that the medical officer should be supreme, since he is not a house physician with a visiting staff, but is responsible both for the treatment and management of the patients. For example, Dr. C. E. S. Fleming and others are anxious to introduce graduated labour, but how is this possible until the medical officer has control of the outdoor servants? At Winsley, moreover, the secretary is not resident. The amendments, proposed by Dr. Fleming with a view to securing the supreme control to the resident medical officer, have been defeated, and the report of the consultative medical board, which included a previous resolution that they "regret to find that by a recent alteration of the rules the status of the medical officer is no longer that of a medical superintendent of the institution," has not been adopted by the board of management. This reveals a serious division of opinion, and implies a practice at variance with the custom and well-being of the best sanatoria.

### A MENTAL SPECIALIST AND FAMOUS TRAILS.

Dr. Lyttleton Stewart Forbes Winslow, who died suddenly last Sunday at the age of seventy, was not merely a noted authority on insanity, but recognised by the public from the part he played in connection with many famous trials during the latter quarter of the nineteenth century. He graduated in medicine at Cambridge in 1870, and soon assisted his father, Dr. Forbes Benignus Winslow, in his large mental practice, and for twenty years saw much work in private mental hospitals. At the end of this period he had begun to appear as an expert witness in many famous trials not only in England but America, which thus occupied him while practising in his special subject. In 1877 he was instrumental in securing the reprieve of the four persons who had been sentenced to death for the murder of Mrs. Staunton by starvation. In 1878 he was called in to examine the state of mind of the Rev. Mr. Dodwell, who fired at Sir George Jessel, the then Master of the Rolls. Among the most famous criminal cases in which his experience was invoked were those of Mrs. Maybrick, the baby farmer Mrs. Dyer, and the man who was convicted of the murder on the Brighton line. Institutional workers will probably recall that the British Hospital for Mental Disorders in North London was founded by him. His main interests are well reflected in the thesis on "The History of Lunacy Legislation," for which he received the degree of D.C.L. from Oxford, and that on "The Criminal Responsibility of the Insane," which embodied the researches which gained him the Cambridge LL.D. He also published a work on the therapeutic value of hypnotism in mental disorders. His "Handbook for Attendants on the Insane" should also be mentioned.

### PENALISING RESPECTABILITY.

Our attention is drawn to a letter of recommendation issued by the Southern Dispensary, Bath, in which appeared among the suggestions of the committee to subscribers for the disposal of their letters to applicants a direction ". . . to avoid as far as possible giving them to persons whose dress and general appearance indicate their capability of paying for medical advice . . ." Such a suggestion is inevitably made to many inexperienced persons who may be tempted to judge the worthiness of a case by outward appearance. To do so is nothing short of penalising respectability in the case of some, or saving from degradation those whom dispensaries are established to help in the case of others. The truth is no directions are of any avail. Each case must be judged on its merits. That is why an attempt to make hospital or dispensary subscribers the judges is to court disaster. The petty patronage on which the letter system is based is a very bad one for patients.

### LEASEHOLDS AS A HOSPITAL INVESTMENT.

A difference of opinion amongst the managers of the Grimsby Hospital as to the policy of investing the hospital funds in leaseholds was evidenced at a recent meeting of the management board, when a recommendation of the finance committee that a rule should be altered so as to preclude the investment of funds in leaseholds was rejected by the board. A member of the board, Mr. T. Mountain, expressed surprise that the suggestion should have been made, as it was impossible to find other securities than leasehold in Grimsby, and, inasmuch as they had the protection of exceedingly shrewd men and could get their securities re-valued from time to time, there was no fear of loss arising. Another member of the board, Mr. C. F. Carter, expressed a similar view, and said that so long as there were guarantees as to the length of the lease, and proper valuation, investment in leaseholds was perfectly safe. In the result a resolution by Mr. Mountain that the rule as it stood should be recommended for confirmation by the proper committee, and that a re-valuation of property be made at intervals of not less than ten years, was carried. As we do not appear to have received a report of the Grimsby Hospital for some years, we have no information as to whether the investments referred to are mortgages on leasehold property or actual leases to the trustees. It is not easy to find investments for charitable funds which yield a good return, and no doubt there is much common sense in the views expressed by Mr. Mountain and his colleague; but it would be interesting to know whether any provision is made by way of a sinking fund to replace capital at the expiration of the leases.

### THE MENTAL DEFICIENCY BILL.

The innate cynicism of the politician who is only a politician was surely never shown with more naïveté than was displayed the other night by Mr.

Pringle, the Liberal member of Parliament for North-West Lanarkshire, in his appeal to the Government to drop the Mental Deficiency Bill. Other Liberals have opposed the Bill: Mr. Handel Booth thinks it "anti-Christian," and Mr. Wedgwood talks of the proposed restraint of the feeble-minded as an "awful doom." We may not agree with these estimates of the results of the Bill, but at least they have an ethical foundation. But what is Mr. Pringle's argument against it? That it would make electioneering capital for the Opposition! This Bill was "deck cargo" and might swamp the ship! When asked by another Liberal member what were the measures in favour of which he wished to jettison a useful cargo like this Bill, Mr. Pringle replied "Home Rule and Welsh Disestablishment." Looked at dispassionately, it can hardly be disputed that it is more essential that the coming generation should be born of sane and healthy parents than that they should be born under one form of Government or another, either of which will give them equal chances of development, or that one branch or another of the Christian Church should have a prestige and pre-eminence over another. These are superficial wounds that smart sharply but do not kill. But the health and brain of a nation are a vital matter. Defectives, be they Church or Chapel, Nationalist or Unionist, are a weakness and ultimately a danger to the State. The Empire may thrive under a tyranny of Church or State, but never without health of mind and body. But all this matters nothing to Mr. Pringle. He admits that the case of the feeble-minded is a cruel one, but he offers no suggestion as to how it is to be bettered. He thinks there is too much compulsion in the Bill—as if compulsion would ever be used to place defectives in institutions if they were being properly looked after at home! If the House of Commons is wise it will listen to the opinions of the few—too few—medical men that belong to it, rather than to those of the professional politician, like Mr. Pringle, who sees in every measure only a more or less judicious vote-catching device.

### TENNYSON'S DOCTOR.

The sudden death of Dr. George Henry Roqué Dabbs removes an interesting medical personality. Known chiefly perhaps as Tennyson's doctor, he was for long a well-known figure in the Isle of Wight. In the Memoirs of Lord Tennyson, published by his son, Dr. Dabbs' name is several times referred to. A medical graduate of Aberdeen and a student of King's College Hospital, he travelled as a young man in France and Spain and then settled down, apparently for ever, in the Isle of Wight. In Tennyson's last illness he was in constant attendance. In 1903, however, he left the Isle of Wight and settled down to a consulting practice in the City. He was also a frequent contributor to health and sporting magazines, published several books, including a few plays, and amused himself by issuing a curious periodical called *My Journal*, to which he was the sole contributor. His short, incisive name seemed to fit in well with his small stature, quick gestures, and marked mannerisms.

### A LONDON HOSPITAL SECRETARY RESIGNS.

Mr. William Hedges, who has just celebrated his golden wedding, has been compelled to resign the post of honorary secretary of the King Edward Memorial Hospital and Dispensary at Ealing. He has filled the post for ten years, having been appointed in 1903 successor to the present Chairman of the hospital, Alderman Joseph Box. Pending the next meeting of the general committee, the duties are being discharged by Captain R. R. Kimmitt, who has acted as assistant secretary for some little time.

### COLCHESTER'S NEW MENTAL HOSPITAL.

The new Essex and Colchester Mental Hospital, Myland, may be said to have started work at the beginning of this month, the patients having been admitted during the last week in May. The medical superintendent is Dr. R. C. Turnbull. Six years have been spent on the buildings, which have been erected from the designs of Mr. F. Whitmore, the county architect, and Mr. W. H. Town, A.R.I.B.A., and which will have accommodation for two thousand patients. The main block is virtually completed. Some fifty attendants will be on the staff, and the various units of the entire institution include a model farm, infectious, phthisical, observation, and idiot children's blocks. The district served by the institution includes those parts of Essex which do not now rely upon the Brentwood institution. The total cost of land, buildings, and equipment is reckoned at nearly half a million.

### "MEDICAL HOMES FOR PRIVATE PATIENTS."

The recent article giving a patient's experiences in a nursing home and in the pay ward of a voluntary hospital brought to light how much at the mercy of their ignorance patients may be in selecting a private nursing home, sanatorium, or health resort. The medical practitioner, though for different reasons, is often in need of assistance in making choice of an institution to suit the requirements of individual patients. To both medical men and their patients the Classified Directory, with lists of medical consultants, which is published by The Scientific Press, Ltd., 28-29 Southampton Street, Strand, W.C., price sixpence, and entitled "Medical Homes for Private Patients," is an invaluable guide. It tells the patient where to be ill, not at all an easy question for a layman to decide without help, and gives to the practitioner at a glance the choice open to him in the matter of special institutions. The present, or eighth, edition has so nearly attained its ideal of completeness that the Editor would be glad to receive notice of any omission, which, with particulars of all new institutions, should be sent to the Editor, "Medical Homes," c/o The Scientific Press, Ltd., 28-29 Southampton Street, Strand, W.C.

### THE SPARE FUNDS OF INSURANCE COMMISSIONERS.

Owing to the number of insured persons in London who have not yet chosen their panel doctor a sum of £34,000 is in hand, and recent discussion took place as to how it should be distributed. One of the more reasonable suggestions was that it should be divided between those panel doctors who had fewer than two thousand patients upon their panel, but, taking fright at the possibility of any distribution being illegal, it was decided to make no distribution at present. In the meantime three more suggestions for allocating any spare funds at the disposal of the Insurance Commissioners have been put forward by a medical man. The first is to augment the incomes of those hospitals in which insured persons have been treated, the second is to aid the funds of the district nursing associations, and the third is to establish or assist existing seaside convalescent homes for cases other than tubercle. The doctors appear to have a *prima facie* claim, which they are not slow to enforce by asserting that they only accepted the rate which they did accept because that rate would apply to all insured persons, irrespective of health or illness. To this it is objected, however, that the rate was accepted by them as applying to all insured persons on their list, a somewhat different matter, as the numbers of insured persons who have not yet chosen a panel doctor has made plain. In the matter of these rival claimants it would be interesting to have the hospitals' views on this matter.

### THE PROVISION OF SANATORIA FOR LONDON.

Mr. R. W. Walden, chairman of the Metropolitan Asylums Board, will have many sympathisers in his concise presentation of what the Board has done to prevent sanatorium provision under the Insurance Act from being a fiasco in London. Being technically a Poor-Law authority, it was expressly excluded from the Act, and a roundabout arrangement had to be invented by which the London Insurance Committee made arrangements for sanatoria with the County Council, which in turn arranged with the Board. In February, under this three-cornered arrangement, the Downs Sanatorium, Sutton, was converted and opened for patients. It can accommodate 350 patients. Later, part of the Northern Hospital, Winchmore Hill, was opened with accommodation for 200 patients. So far, Mr. Walden states, 602 patients have received treatment under the Board, and of these, apart from discharges and deaths, 236 patients were in the Downs Sanatorium and 144 in the Northern Hospital last week. When the day comes to write the history of Insurance legislation and its application in England, the work of the Metropolitan Asylums Board in supplying sanatoria accommodation for London will have its own place, for the work which it has done has been undertaken and is now being carried on without any *locus standi* and in a position that can be rectified only by the disastrous expedient of removing the patients from its care, or, alternatively and sensibly, by recognising that it is not a Poor-Law authority in the sense which led the framers of the Act to exclude such from arrangements for receiving sanatoria patients. Its work outside the Poor Law is as large as, if not larger than, within.

### INSURANCE AND SANATORIUM TREATMENT.

The Editor of the *Medical Press* takes us somewhat severely to task for certain comments which we offered a fortnight ago on a cartoon in the *Westminster Gazette.* We made the statement that the prevention of tuberculosis, as opposed to its cure, is not much advanced by the Insurance Act; and the *Medical Press* thinks we forgot the influence of sanatorium benefit in isolating the sufferers from tuberculosis, and so preventing them from spreading the disease. We should like to assure the *Medical Press* and our readers that this point was not overlooked, and was not absent from our minds when the sentence was written. Broadly speaking, those who are received into sanatoria under the Act are *not* the infectious cases; but those in early stages, mostly with "closed tubercle," who are, in fact, not a source of danger to their neighbours and relatives. The advanced cases, expectorating millions of tubercle bacilli *per diem*, which really are a source of danger and infection, are not taken into sanatoria, but remain at home under what is often, as we can vouch, very perfunctory treatment from a panel practitioner. Moreover, even those who do get allowed sanatorium treatment have, as a rule, to wait six weeks for it, often longer, during which time they are left pretty much to their own devices: that, at least, is what has happened in London within our own knowledge. The further accusation of attempting to make party capital out of the Insurance Act leaves us unmoved: surely the *Medical Press* admits that it is just what the *Westminster Gazette* was doing, and what we protested against? Finally, the Editor of the *Medical Press* misquotes us very seriously. In his closing remarks he ascribes to us the statement that the Insurance Act no whit advances the prevention of tuberculosis; whereas, what we said was that Insurance sanatoria do not do so. And he then says we fail to grasp the true intention of the Act; whereas all we did was to comment on its effects as administered, not its intentions.

### THE LATEST THING IN HOSPITAL FETES.

A remarkable fête was held at the Albert Hall on Wednesday and Thursday this week in aid of the London Hospital's quinquennial appeal. Three members of the committee, Mr. Coke, Mr. Goetz, and Mr. Goschen, have taken immense pains to make it a success, and the novelty of its idea is seen in its title—namely, "The Noah's Ark Great Fair and Variety Show." A representative number of West End firms have undertaken to hold stalls in the fair, and pay a premium for the privilege of having their names appear as supplying the stall. The original feature which differentiates this from the ordinary bazaar is that saleable goods are furnished at moderate prices. The purchaser is intended to get his money's worth, and the firms are to give a substantial percentage towards the funds. A dress parade of mannequins foreshadowing the Ascot fashions was one original idea! In the centre of the hall an elaborate Japanese garden was exhibited, and the stalls were guarded by antediluvian animals, which were designed to form a contrast to the up-to-dateness of the wares. The pavilion for American drinks and candies was another original idea, and society ladies were among the stall-holders. In addition to numerous side-shows, theatrical performances were given by prominent actors, and Madame Kirkby Lunn arranged to take part in the concerts which were held on both days.

### AMENDING THE INSURANCE ACT.

The date is rapidly approaching when the sincerity, or otherwise, of the Government's intentions to amend the Insurance Act can be tested by their actual proposals. The last irony attending the disastrous progress of the Act from its first inception until now is seen in the fact that hardly have the Government announced their intention to amend it when the Admiralty has to make a further claim on the Exchequer, thus absorbing at once any otherwise available funds. Apart altogether from the views of the friendly societies and the mere mechanical, though important, changes expected in changing the date for the make-up of the Insurance cards from the quarter to the half year, the proposals of the British Medical Association certainly involve expenditure. Among the changes asked for are that the doctors should have power to make a small charge for night calls; that the Government should provide a special fund, apart from that already allocated to medical benefit, to pay for the treatment of insured persons when away from home on the ground of ill-health; and that the Commissioners should appoint medical referees, with reasonable security of tenure, to decide on cases of alleged malingering.

### GLASGOW'S NEW PROFESSOR OF MEDICINE.

On the recommendation of the Secretary of State for Scotland the King has approved the appointment of Professor Thomas Kirkpatrick Monro, M.D., to the Professorship of Practice of Medicine in the University of Glasgow. Professor Monro, who succeeds in his new appointment the late Professor Gemmell, is at present Professor of Medicine and Dean of the Medical Faculty in St. Mungo's College, Glasgow. Formerly a student at Glasgow, he has had a long connection with the city, having held many hospital appointments there, and being now a member of the Medical Advisory Board of Consumptive Sanatoria of Scotland. He has issued numerous publications on medical topics and a manual of medicine which has run into several editions.

### NEW POST FOR A LONDON SUPERINTENDENT.

At a meeting of the Council of Almoners of Christ's Hospital, presided over by Alderma Sir Joseph Savory, Mr. Gerald E. Friend, superintendent and resident medical officer to St. George's Hospital, was elected resident medical officer. Mr. Friend, so well known to St. George's men, graduated in Arts at Oxford, and has held the post of superintendent for six years. He succeeds at Christ's Hospital Dr. W. Aldersmith, who retired recently after forty-two years' service. Mr. Friend is a surgeon-captain in one of the battalions of the Royal West Surrey Regiment, and, in case the title of his post at St. George's sounds ambiguous to those who do not know the facts, it may perhaps be added that the superintendent and resident medical officer at St. George's virtually corresponds to the medical superintendent of those hospitals which have as administrative chief a medical man.

### MARRIAGE IN A HOSPITAL CHAPEL.

The use of a hospital chapel for a marriage service is sufficiently uncommon to be worth recording. On Wednesday last week the Chapel of the Royal Alexandra Infirmary, Paisley, was used for this purpose when Dr. Robert McCowan Hill, a former house surgeon, was married to Miss Alberta Brooke Jordan, senior sister at the infirmary. The patients as well as the directors of the infirmary gave presents, and the reception was held at the Nurses' Home after the ceremony, the Matron, Miss Alexander, acting as hostess.

### A HOSPITAL BEQUEST REVOKED.

More than usual interest attaches to the testament of Sir Richard Nicholson, of Banff, and of Hyde Park, in whose will are thirteen codicils. He has revoked a bequest to the Chalmers Hospital, Banff, "in consequence of the depreciation in value of my brewery Debentures by reason of the predatory Licensing Bill of the Government," in a codicil dated November 24, 1908. Sir Richard Nicholson had become blind during the last four years, and it is interesting to note that his charitable bequests include a sum varying with the amount of his residuary estate, but not exceeding £12,000, to the Ophthalmic Hospital, Moorfields. Provision is also made for a like sum to that hospital, any hospital or institution at Aberdeen, for the welfare of the blind, or any hospital, home, or institution for the blind in the county of Middlesex, and any hospital, home, or institution for convalescents in Middlesex, Herts, Kent, Surrey, Hants, or Sussex, as his executors may select; a like sum to his wife, with the request, but without creating any trust, that she will use it for the purposes already indicated by him, the balance being devoted to the Ophthalmic Hospital, Moorfields. He also left £1,000 to the Chalmers Hospital, Banff, during the life of Dr. Ferguson, to pay the income to him to be used by him as he may think fit for the benefit of the patients.

### THE HOSPITAL PARCEL.

The quinquennial appeal of the London Hospital has been aided somewhat by an anonymous gift of £10,000, which appeared in the form of a brown paper parcel addressed to Mr. E. W. Morris, the secretary, and which—such is the state of anxiety induced in responsible people by the dynamitard tactics of certain young ladies—he thought might perhaps be a bomb. The money was in the form of bearer bonds, and is the largest anonymous sum ever received by the London Hospital. In the gratitude engendered by such a useful " find " as this it seems almost superfluous to inquire into the motives of anonymous givers; but surely they are of no less interest than those which lead others to give with publicity? In the present case the anonymity seems to be complete, for no instructions were left, as is sometimes the case, for acknowledgment to be made in any particular way. We have known wealthy people call upon a hospital dressed as very humble folk, and give as it seemed purposely an infinite amount of trouble to the officials before taking their departure. But afterwards, when courteously treated, they have sent as much as £10,000, and subsequently expressed gratification with the management on account of the courtesy extended to themselves. To a hospital secretary it does not matter greatly whether the brown paper parcel on his desk is taken for a bomb or bonds, providing the money is found inside. We expect such a bomb containing many thousands of pounds with a proper clock adjustment will yet find its way on to the secretary's table of some voluntary hospital in due course.

### REMUNERATION OF TUBERCULOSIS OFFICERS.

The machinations of the National Insurance Act have already resulted in increases of salaries to officers directly associated with its administration, especially to those connected with the control of tuberculosis dispensaries. Only last December the Deptford Borough Council opened a dispensary, when they appointed Dr. William Scarisbrick, M.D., D.P.H., tuberculosis officer and assistant medical officer of health. His salary was £300 a year, rising by two annual increments to £350. Dr. Scarisbrick has resigned the appointment, having been selected medical superintendent of the Benenden Sanatorium in Kent. Since the appointment of Dr. Scarisbrick at Deptford, other Metropolitan Borough Councils have appointed similar officers at increased remuneration, and consequently the public health committee at Deptford has recommended and the Council agreed that in the future the remuneration of the tuberculosis officer shall be a commencing salary of £300 a year, rising by four annual increments to £400.

### THE AGE OF SURGERY.

As a memorial to the late King, a new operating theatre has been opened at the Tynemouth Victoria Jubilee Infirmary, North Shields, by Sir Thomas Oliver. Mr. George Baird, the president of the infirmary, welcomed the visitors, and accepted the theatre on behalf of the governors as a gift from the subscribers, who had raised over £700. Sir Thomas Oliver gave an address in which he described the present as the age of surgery, and compared its advance with the smaller progress of medicine, adding that " the results obtained by surgery certainly appeal far more to our minds than the success gained in the wider fields of medicine." This statement seems to us too loosely phrased to be convincing. It is no doubt easier to make the public grasp the success of surgery than the more scientific success of an extension of our knowledge of medicine, but a surgical success does not " appeal to our minds " so much as to the cruder sensation of amazement. The element of a fine dexterity is necessary to a successful surgeon. A fine and painstaking mentality—that is to say, pure science—is the basis of advance in medicine. Is not surgery honoured enough without claiming for it the merits of the sister science?

### A LOSS TO GUY'S HOSPITAL.

Only a few weeks ago St. Bartholomew's lost one who promised to be a tower of strength to his hospital and school in Mr. Etherington-Smith. Now it is Guy's turn to mourn their obstetric surgeon and lecturer, Mr. J. H. Targett, who died on May 27 after a long illness. It has been known for months that Mr. Targett's health was very precarious. The late surgeon was, or had been, on the staff, not only of Guy's, but also of the Bolingbroke, Samaritan, and Evelina Hospitals. He was a Fellow of the Royal College of Surgeons, and had also been Erasmus Wilson Lecturer. The vacancy thus created at Guy's Hospital illustrates strongly the curious extent to which luck enters in connection with hospital staff appointments. It is common knowledge that, until Mr. Targett's illness began, the prospects of promotion in this speciality appeared so remote that two brilliant young surgeons in turn gave up the " waiting game " in despair, and transferred their energies elsewhere. Now, however, the unexpected has happened, and possibly both of them are wishing they had taken the risk of an apparently unending wait. Guy's Hospital, however, is fortunate in the quality of its young gynæcologists, and can doubtless supply a man for the vacancy who will maintain the high traditions of the institution.

### THIS WEEK'S DRUG MARKET.

Several changes in value have taken place during the week, but few of them have been of special importance. Following on reports from the growing districts as to the favourable condition of the opium crop there has been a downward tendency in the price of this drug, and in consequence makers of morphine and codeine have reduced their quotations. There appears, however, to be some difficulty in effecting shipment from Smyrna, so that the value of opium on the London market is higher than should be the case in view of the favourable reports. The position of cod-liver oil is unchanged. A good business has been done in asafetida at lower prices. Chloral hydrate is slightly dearer. Oil of cloves has been slightly reduced in price as a result of a fall in the value of cloves. Essence of lemon continues to advance in price. Oil of orange is also dearer. Oil of bergamot is cheaper.

